# Inhibition of GSDMD Activates Poly(ADP-ribosyl)ation and Promotes Myocardial Ischemia-Reperfusion Injury

**DOI:** 10.1155/2022/1115749

**Published:** 2022-06-24

**Authors:** Zheng-hao Zhang, Zi-guan Zhang, Min-wei Chen, Ying Yang, Run-jing Li, Jia-jia Xu, Cui Yang, Yu-ying Li, Hong-wei Chen, Shi-xiao Liu, Yan-ling Li, Ping Luo, Yi-jiang Liu, Wen-bo Chen, Zhong-gui Shan, Zheng-rong Huang

**Affiliations:** Department of Cardiology, Xiamen Key Laboratory of Cardiac Electrophysiology, Xiamen Institute of Cardiovascular Diseases, The First Affiliated Hospital of Xiamen University, School of Medicine, Xiamen University, Xiamen, China

## Abstract

The precise control of cardiomyocyte viability is imperative to combat myocardial ischemia-reperfusion injury (I/R), in which apoptosis and pyroptosis putatively contribute to the process. Recent researches indicated that GSDMD is involved in I/R as an executive protein of pyroptosis. However, its effect on other forms of cell death is unclear. We identified that GSDMD and GSDMD-N levels were significantly upregulated in the I/R myocardium of mice. Knockout of GSDMD conferred the resistance of the hearts to reperfusion injury in the acute phase of I/R but aggravated reperfusion injury in the chronic phase of I/R. Mechanistically, GSDMD deficiency induced the activation of PARylation and the consumption of NAD^+^ and ATP, leading to cardiomyocyte apoptosis. Moreover, PJ34, a putative PARP-1 inhibitor, reduced the myocardial injury caused by GSDMD deficiency. Our results reveal a novel action modality of GSDMD in the regulation of cardiomyocyte death; inhibition of GSDMD activates PARylation, suggesting the multidirectional role of GSDMD in I/R and providing a new theory for clinical treatment.

## 1. Introduction

Acute myocardial infarction (AMI) represents a major health problem worldwide, due to its high morbidity and mortality [1, 2]. Recanalization of the coronary has been shown to benefit the prognosis of patients with AMI, but paradoxically, reperfusion itself often causes irreversible damage to the myocardium and coronary circulation and even increases infarction size, called ischemia-reperfusion injury (I/R) [3]. These manifestations are mainly attributable to the progressive cardiomyocyte death caused by inflammatory responses from ischemia and hypoxia [4]. In the acute phase, tissue hypoxia directly causes cardiomyocyte death. After blood flow is restored, inflammatory cytokines are released and extracellular matrix synthesis is increased to repair collagen scaffolders. In the chronic phase, inflammation leads to further death of cardiomyocytes at the border of infarction, while excessive fibrous tissue repair leads to pathological myocardial remodeling and heart failure [5].

More and more studies have shown that the death pathway of cardiomyocytes is not simple necrosis but has more complicated patterns, including apoptosis, necroptosis, autophagy, and pyroptosis [6–9]. Pyroptosis is an inflammatory form of death [10]. It activates caspase-1 via inflammasome, and the activated caspase-1 cleavages the substrate GSDMD, whose N-terminal forms pores in the cell membrane, resulting in cell rupture and IL-1*β* release [11, 12]. Previous studies have confirmed that pyroptosis is correlated with the pathological processes of AMI and I/R [13, 14].

On the other hand, widespread studies have proved that apoptosis persists for hours to weeks after I/R, but there is a delicate balance in the mode of its activation [15]. Early apoptosis mostly occurs at the border zone of infarction to remove cardiomyocyte DNA damage, the level of late apoptosis gradually decreases, and long-term apoptosis can significantly promote heart failure [15–17]. PARP-1 is a repair enzyme that promotes DNA repair by poly(ADP-ribosyl)ation (PARylation). However, under the condition of inadequate energy supply, excessive PARylation leads to depletion of NAD^+^ and ATP and finally causes apoptosis [18].

Gasdermin D (GSDMD), as the executive protein of pyroptosis, is mainly capable of forming pores on the cell membrane and releasing IL-1*β*, leading to cell death and inflammation [19]. Recent studies have shown that GSDMD plays a key role in myocardial I/R, and inhibition of GSDMD can significantly reduce myocardial injury induced by pyroptosis, but the effect of GSDMD on other forms of cell death is currently unknown [20]. In this study, we demonstrated that GSDMD inhibition did indeed reduce myocardial injury in the acute phase of I/R by blocking pyroptosis but significantly activated PARylation through its interaction with PARP-1 and further worsened the cardiac function by promoting apoptosis of cardiomyocytes.

## 2. Methods

### 2.1. Animals

Animal experiments were managed by the Laboratory Animal Management Ethics Committee of Xiamen University. The GSDMD^−/−^ mice on C57BL/6 background were donated by Jiahuai Han Laboratory. Male mice of 8 to 12 weeks were used in the experiments.

### 2.2. Cell Culture and Hypoxia/Reoxygenation Injury

H9C2 cells were maintained in high glucose DMEM supplemented with 4 mM glutamine, 10% FBS, 100 IU/mL penicillin, and 100 *μ*g/mL streptomycin at 37°C in 5% CO_2_.

For hypoxia/reoxygenation experiments, cells were transferred to a closed hypoxic apparatus (filled with a mixture of 95% N_2_, 4% CO_2_, and 1% O_2_) and deprived of glucose and FBS in the medium for 4 hours, 6 hours, or 12 hours, followed by returning to normal oxygen and complete medium for 2 hours. Cell treatment was performed before hypoxia by adding PJ34 (2 *μ*M for final concentration, Sigma-Aldrich, USA).

### 2.3. I/R Protocol

Adult male mice were anesthetized and connected to the small rodent ventilator with endotracheal intubation. The chest hair was removed with depilation cream; the skin was cut at the left 3rd intercostal area. The heart was exposed through a thoracotomy, the left anterior descending coronary artery was ligated with silk thread (6-0), and the heart was temporarily externalized through the left thoracic incision. The ligation was unwound 45 minutes later, and blood flow was restored for 4 hours, 24 hours, or 4 weeks. Mice in the sham group received the same operation except for silk ligation. Mice in the treatment groups were injected with PJ34 (2 mg/kg/d, Sigma-Aldrich, USA) through the caudal vein. The same amount of normal saline was given to the control group. The mice were sacrificed 4 hours, 24 hours, or 4 weeks after the operation, and the samples were collected for relevant experiments.

### 2.4. Myocardial Infarct Size Measurement

After anesthesia, the chest of mice was opened, and the remaining 6-0 silk thread was ligated again. The heart was perfused with 1% Evans blue, washed with normal saline, and frozen at -80°C for 5 minutes. Then, the part below the ligation line was cut into 4 parallel slices, stained in 1%TTC for 10 minutes, and then stored in 4% paraformaldehyde. After taking pictures, the myocardial infarct area was calculated by ImageJ 1.52a.

### 2.5. Masson's Trichrome Staining

Mice were sacrificed after I/R (45 min/4 w), their hearts were fully lavaged, and then, myocardial infarction tissue was dissected on the parallel ligation plane, and the tissue was fixed, dehydrated, and paraffin-embedded. The heart tissue was sliced and attached to the glass slide, restored moisture, and then stained. Masson's trichrome staining kit (D026-1-3, Nanjing Jiancheng, China) stained fibrotic tissue. An intelligent stereo microscope (Leica M165FC, Germany) was used to take micrographs at ×2 magnification. The collagen staining area (blue) as a percentage of the total cardiac area was calculated as fibrosis volume fraction.

### 2.6. Echocardiography

The VEVO2100 ultrasound imaging system (VisualSonics, Canada) was used to assess heart function. Mice were continued to inhale 1-2% isoflurane and removed chest hair with surgical hair removal cream. Echocardiographic images were measured in M mode. EF (Ejection Fraction), FS (Fractional Shortening), LVIDs (Left Ventricular Internal Diameter at end-systole), and LVIDd (Left Ventricular Internal Diameter at end-diastole) were obtained by measuring the marker lines, and the images and data were saved.

### 2.7. shRNA Interference-Mediated Gene Silencing

GSDMD (shGSDMD1: 5′-GGAGATCATGCAACGTGAAAG-3′ and shGSDMD2: 5′-TGCCTCCATGAATGTGTGTAT-3′) and PARP-1 (shPARP-1: 5′-GGAAGTGAAAGCAGCCAAT-3′) shRNA plasmids were purchased from GenePharma (Shanghai, China). To generate stably transfected cells, shRNA plasmids packaged with lentivirus were used to infect the cells. Stable cell lines were screened by puromycin.

### 2.8. LDH Release

Mouse serum and cell supernatant were collected to detect LDH release. LDH was detected using the assay kit from Beyotime Biotechnology (C0017, China). OD_490_ was detected to evaluate LDH release.

### 2.9. Cell Viability Assays (MTT)

Cells were inoculated into 96-well plates at 2 × 10^4^ cells per well. After hypoxia and reoxygenation for 6 h/2 h or 12 h/2 h, 10 *μ*L of MTT solution (5 mg/mL) was added to each well and incubated at 37°C for 4 h, then dissolved overnight with 10% SDS (containing 0.01 M HCl) 100 *μ*L. OD_560_ was detected to evaluate cell viability.

### 2.10. Flow Cytometry for Apoptosis

Cells were treated with the assay kit from Yeasen Biotechnology (40304ES60, China) and analyzed by flow cytometry (Beckman Coulter, USA). Isotype controls were used to set appropriate gates. Data were analyzed with CytExpert (Beckman Coulter, USA) and FlowJo X10 (Becton, Dickinson and Company, USA). For all samples, 10,000 cells were analyzed for plot generation.

### 2.11. Western Blot

The heart tissue and H9C2 cells were ground and lysed by protein lysis buffer (Beyotime, China) with PMSF and phosphatase inhibitor cocktail. Total protein samples were extracted from the lysate, and the protein concentration was detected. The sample was loaded into 8% or 12% SDS-PAGE gel for electrophoresis and then transferred to the PVDF membrane. It was blocked for 1 hour and then immersed in primary antibody for 12 hours at 4°C, such as GAPDH (Proteintech, 60004-1-Ig, USA), GSDMD (Abcam, ab219800, ab209845, UK), NLRP3 (Abcam, ab214185, UK), caspase-1 (Abcam, ab179515, UK), IL-1*β* (Abcam, ab9722, UK), caspase-3 (Cell Signaling Technology, 14220T, USA), caspase-7 (Cell Signaling Technology, 12827T), caspase-8 (Beyotime, AC056, China), Bcl-2 (Beyotime, AB112, China), Bax (Beyotime, AB026, China), PARP-1 (Cell Signaling Technology, 9532S, USA), and poly/mono-ADP ribose (Cell Signaling Technology, 83732S, USA). The membranes were washed with TBST twice, immersed in the secondary antibody for 1 hour, and then washed again. Bands were displayed by the ECL detection kit (Millipore, USA).

### 2.12. Coimmunoprecipitation

To perform coimmunoprecipitation, H9C2 cells were lysed in cold IP lysis buffer. Lysates were incubated overnight with primary antibody or control immunoglobulin G (Ig G), and then, protein-A/G agarose (Fisher Scientific, USA) was added and incubated for 2 hours. The centrifugal precipitates were washed three times with IP lysis buffer and then used for western blot.

### 2.13. NAD^+^ and ATP Assays

NAD^+^ measurement was performed by the NAD^+^/NADH assay kit (Beyotime, S0175, China), which is based on a color reaction of WST-8. The amount of NAD^+^ in cell samples was detected by a microplate reader based on the operating guidelines.

ATP measurement was performed by the enhanced ATP assay kit (Beyotime, S0027, China), which is based on the light produced by ATP-dependent luciferase. The amount of ATP was detected by a GloMax 20/20 luminometer (Promega, USA) based on the operating guidelines.

### 2.14. Statistical Analyses

All data were measurement data. Tests for normality and homogeneity of variance were performed. For analysis of differences between two groups, unpaired Student's *t*-test or Welch *t*-test was performed. For multiple groups, one-way ANOVA or Brown-Forsythe was performed, followed by Tukey's test. SPSS 19.0 and GraphPad Prism 9.0 were used to calculate the significance between groups.

## 3. Results

### 3.1. The Pyroptosis Pathway Is Activated in Myocardial I/R

**Figure 1:**
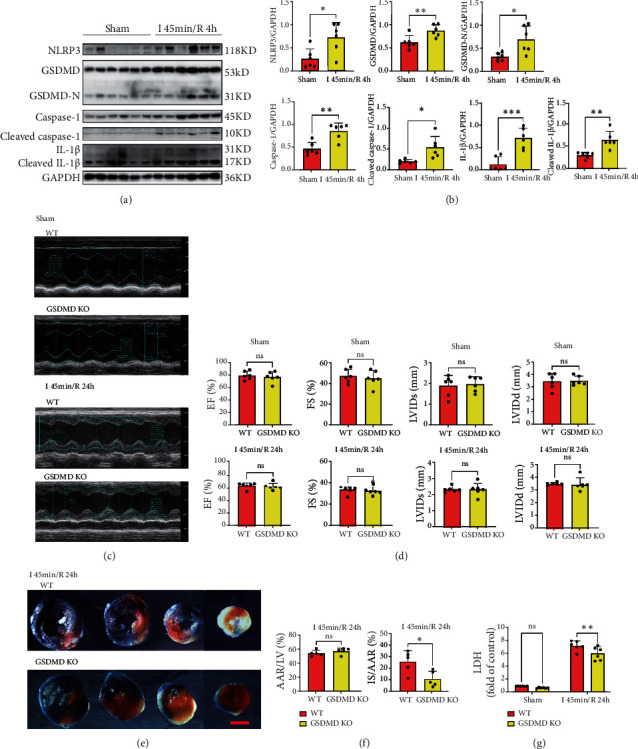
The pyroptosis pathway is activated in I/R, and knockout of GSDMD improves myocardial injury in the acute phase of I/R. (a) Myocardial pyroptosis proteins were detected by western blot. Sham vs. I/R. (b) Quantification of pyroptosis proteins. Sham vs. I/R. (c) Cardiac function was detected by echocardiography. Sham and I 45 min/R 24 h. WT vs GSDMD KO. (d) Quantification of EF, FS, LVIDs, and LVIDd. Sham and I 45 min/R 24 h. WT vs. GSDMD KO. (e) Myocardial infarction size. I 45 min/R 24 h. WT vs. GSDMD KO. Scale bar: 1 mm. (f) Quantification of infarct size. I 45 min/R 24 h. WT vs. GSDMD KO. (g) Serum LDH concentrations. Sham and I 45 min/R 24 h. WT vs. GSDMD KO.

To determine whether the NLRP3/caspase-1/GSDMD pyroptosis classical pathway is involved in the pathological process of cardiac I/R, the I/R model of C57BL/6J mice was used to detect the changes in the proteins associated with pyroptosis. Total protein of the left ventricle was extracted from the mice subjected to 45 minutes of ischemia followed by 4 hours of reperfusion (I 45 min/R 4 h) or sham surgery (sham). Results showed that the levels of NLRP3, GSDMD, GSDMD-N, caspase-1, cleaved caspase-1, IL-1*β*, and cleaved IL-1*β* in the I/R group were significantly higher than those in the sham group. Elevated levels of GSDMD-N and cleaved IL-1*β* indicated activation of GSDMD and IL-1*β* during I/R (Figures 1(a) and 1(b)). These results demonstrated that pyroptosis pathway-related proteins were increased and activated in myocardial I/R.

### 3.2. Knockout of GSDMD Improves Myocardial Injury in Acute Phase of I/R

GSDMD is the core protein of cardiomyocyte pyroptosis, so in our study, GSDMD KO mice were applied to study the potential role of GSDMD in I/R. WT and GSDMD KO mice were randomly subjected to I 45 min/R 24 h or sham surgery, and echocardiography, infarction size, and serum LDH were determined. At the baseline, GSDMD KO mice showed no significant changes in heart morphology or function after sham surgery. After I/R injury, the myocardial infarction size was significantly reduced in GSDMD KO mice compared with the WT mice (Figures 1(e) and 1(f)), and the release of serum LDH was also reduced in the GSDMD KO mice (Figure 1(g)), suggesting that GSDMD knockout inhibited the pyroptosis and reduced the myocardial infarction size during the acute phase of myocardial I/R. The results of echocardiography showed that there was no statistical difference in EF, FS, LVIDs, and LVIDd of WT and GSDMD KO mice after reperfusion for 24 hours (Figures 1(c) and 1(d)).

### 3.3. Knockout of GSDMD Aggravates Myocardial Injury in Chronic Phase of I/R

**Figure 2:**
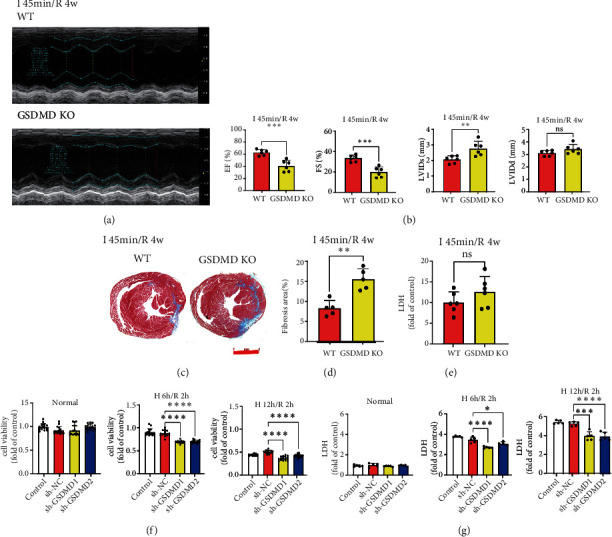
Knockout of GSDMD aggravates myocardial injury in the chronic phase of I/R. (a) Cardiac function was detected by echocardiography. I 45 min/R 4 w. WT vs. GSDMD KO. (b) Quantification of EF, FS, LVIDs, and LVIDd. I 45 min/R 4 w. WT vs. GSDMD KO. (c) Myocardial fibrosis was determined by Masson's trichrome staining. I 45 min/R 4 w. WT vs. GSDMD KO. Scale bar: 1 mm. (d) Quantification of myocardial fibrosis. I 45 min/R 4 w. WT vs. GSDMD KO. (e) Serum LDH concentrations. I 45 min/R 4 w. WT vs. GSDMD KO. (f) H9C2 cell viability was detected in control, sh-NC, sh-GSDMD1, sh-GSDMD2 under normal oxygen, H 6 h/R 2 h, and H 12 h/R 2 h. (g) LDH release of H9C2 cells was detected in control, sh-NC, sh-GSDMD1, sh-GSDMD2 under normal oxygen, H 6 h/R 2 h, and H 12 h/R 2 h.

Within several weeks of coronary reperfusion, the myocardium continues to undergo inflammation and repair, which exacerbate local myocardial injury and irreversible fibrosis [21]. Myocardial fibrosis could lead to reduced compliance, decreased systolic function, and ultimately cardiac remodeling and heart failure [22]. To understand the late effects of GSDMD on myocardial I/R, echocardiography was used to assess cardiac function, and Masson's staining was used to assess myocardial fibrosis in I 45 min/R 4 w. Our results showed that EF and FS decreased and LVIDs increased significantly in GSDMD KO mice after reperfusion for 4 weeks (Figures 2(a) and 2(b)). Meanwhile, Masson's staining showed that the range of myocardial fibrosis in GSDMD KO mice was significantly increased (Figures 2(c) and 2(d)). And there was no statistical difference in serum LDH at the chronic phase of I/R (Figure 2(e)). Contrary to the acute phase effect, results of reperfusion for 4 weeks showed that GSDMD deficiency promoted cardiac injury during the chronic phase of I/R.

To investigate the mode of cardiomyocyte death induced by GSDMD, we further used lentiviral vector shRNA to knockdown GSDMD in H9C2 rat cardiomyocytes to estimate cell viability during the hypoxia and reoxygenation (H/R) process. GSDMD deficiency did not show protective effects on cardiomyocytes during H/R. After H 6 h/R 2 h or H 12 h/R 2 h, the cell viability of sh-GSDMD was significantly decreased than that of sh-NC (Figure 2(f)), while the release of LDH was significantly decreased (Figure 2(g)). This indicated that the cell death was not mainly manifested by cell membrane damage and LDH release, so we speculated that there was an increase in apoptosis in this process.

### 3.4. Inhibition of GSDMD Promotes Apoptosis during I/R

**Figure 3:**
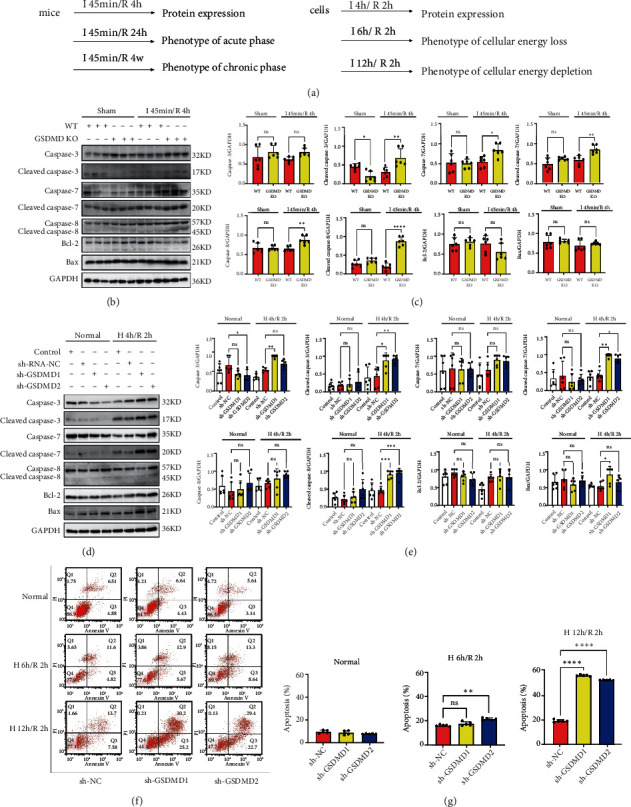
Inhibition of GSDMD promotes apoptosis during I/R. (a) Time diagram of mice and cell experiments. (b, c) Myocardial apoptosis proteins were detected by western blot. Sham and I 45 min/R 4 h. WT vs. GSDMD KO. (d, e) H9C2 cell apoptosis proteins in control, sh-NC, sh-GSDMD1, sh-GSDMD2 under normal oxygen, and H 4 h/R 2 h were determined by western blot. (f, g) Apoptosis of H9C2 cells was detected by flow cytometry. Normal oxygen, H 6 h/R 2 h, H 12 h/R 2 h. sh-GSDMD1 and sh-GSDMD2 cells vs. sh-NC.

We confirmed that GSDMD deficiency protected against myocardial injury in the acute phase of I/R by blocking pyroptosis. However, the heart function of GSDMD KO mice decreased after 4 weeks of final experimental results. In addition, as the main cell death in the I/R chronic phase, apoptosis is a crucial form that cannot be ignored [23], but the effect of GSDMD activation on apoptosis remains unclear. Therefore, we investigated the activation of apoptotic pathways during this process in addition to pyroptosis. Both WT and GSDMD KO mice were performed sham and I 45 min/R 4 h, and left ventricles were taken for detection of apoptosis-associated proteins. It showed that GSDMD KO mice were significantly increased in cleaved caspase-3, cleaved caspase-7, cleaved caspase-8, caspase-7 and caspase-8 than WT mice after I/R, suggesting that GSDMD deficiency activated cardiomyocyte apoptosis during I/R (Figures 3(b) and 3(c)). Similar results were obtained in the H9C2 cell hypoxia and reoxygenation experiment. We found that cleaved caspase-3, cleaved caspase-7, and cleaved caspase-8 increased significantly in sh-GSDMD cells compared with sh-NC cells after H 4 h/R 2 h (Figures 3(d) and 3(e)).

We further examined the effect of GSDMD deficiency on H9C2 cells apoptosis by flow cytometry. The apoptosis degree was detected at H 6 h/R 2 h and H 12 h/R 2 h, and it was found that the apoptosis of sh-GSDMD was significantly increased compared with the sh-NC cells at H 12 h/R 2 h (Figures 3(f) and 3(g)).

### 3.5. Inhibition of GSDMD Upregulates PARylation in Cardiomyocytes

We next sought to identify the intermediate regulatory mechanism of GSDMD deficiency leading to increased cell death in myocardial injury. A number of proteins were found to bind to GSDMD using COIP-MS, among which PARP-1 is considered to be involved in myocardial injury (Figure S3). PARP-1 is a nucleic acid repair enzyme that can repair damaged DNA by PARylation [24, 25].

**Figure 4:**
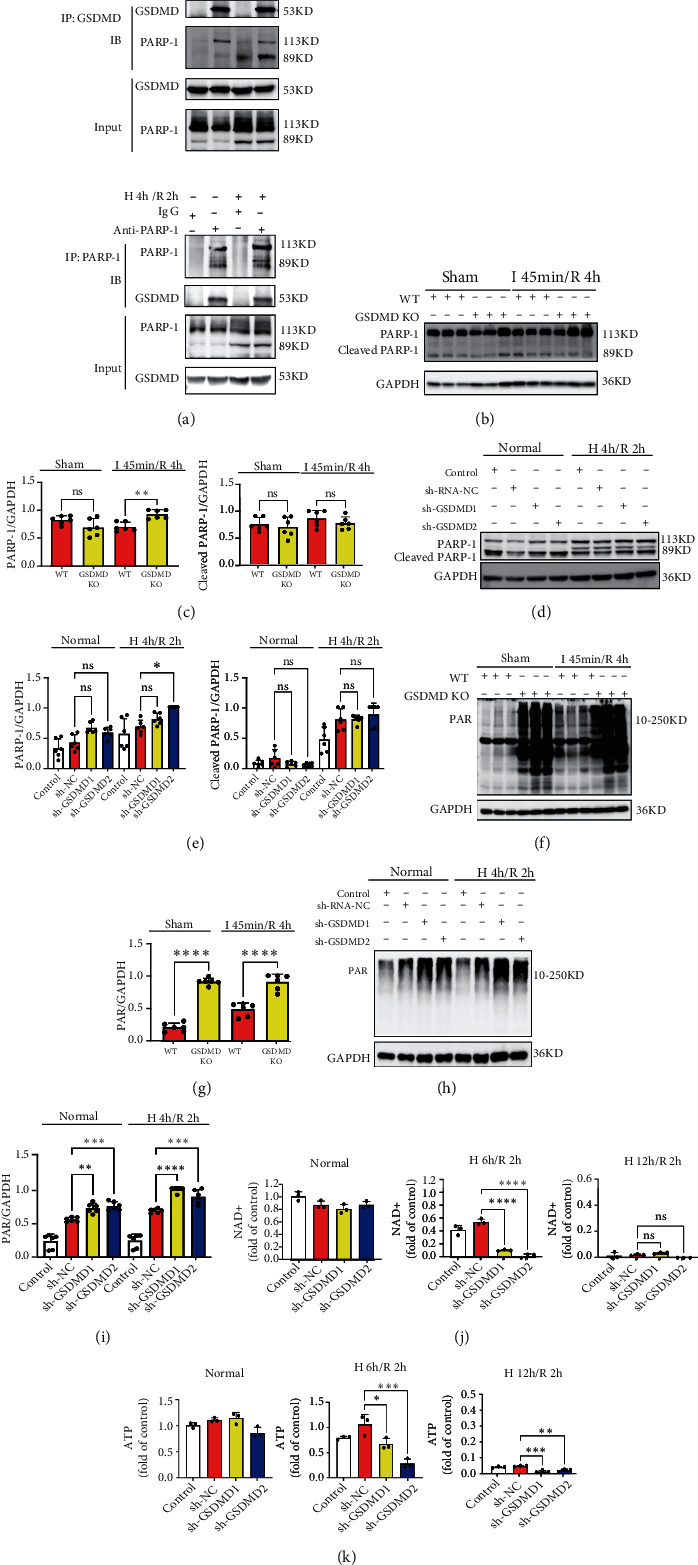
Inhibition of GSDMD upregulates PARylation in cardiomyocytes. (a) Coimmunoprecipitation of GSDMD and PARP-1 in H9C2 cells under normal oxygen and H 4 h/R 2 h. IB: immunoblot; IP: immunoprecipitation. (b, c) PARP-1, cleaved PARP-1, and (f, g) PARylation were detected by western blot. I 45 min/R 4 h. WT vs. GSDMD KO. (d, e) PARP-1, cleaved PARP-1, and (h, i) PARylation in control, sh-NC, sh-GSDMD1, sh-GSDMD2 under normal oxygen, and H 4 h/R 2 h were determined by western blot. (j) NAD^+^ of H9C2 cells was detected in control, sh-NC, sh-GSDMD1, sh-GSDMD2 under normal oxygen, H 6 h/R 2 h, and H 12 h/R 2 h. (k) ATP of H9C2 cells was detected in control, sh-NC, sh-GSDMD1, sh-GSDMD2 under normal oxygen, H 6 h/R 2 h, and H 12 h/R 2 h.

Coimmunoprecipitation assays confirmed the hypothesis that GSDMD and PARP-1 bind and interact with each other (Figure 4(a)).

The most common role of PARP-1 is to be cleaved by caspase-3 during the initiation of apoptosis, and the increase of cleaved-PARP-1 indicates the occurrence of apoptosis [26]. We further explored the correlation between GSDMD and PARP-1. Our results showed that expression of PARP-1 was upregulated in myocardial tissue of GSDMD KO mice after I 45 min/R 4 h, but the cleaved-PARP-1 has no significant changes (Figures 4(b) and 4(c)). H9C2 cells knocked down by shRNA-GSDMD showed similar results after H 4 h/R 2 h (Figures 4(d) and 4(e)).

Previous studies have found that PARP-1 not only participates in apoptosis through cleavage by caspase-3 but also leads to apoptosis and cell death through excessive PARylation in the process of DNA damage [27]. Therefore, we further investigated the changes in protein PARylation and found that both I/R and sham mice with GSDMD deficiency showed significant upregulation of PARylation (Figures 4(f) and 4(g)), and shRNA-GSDMD H9C2 cells showed a significant growth in PARylation after H 4 h/R 2 h (Figures 4(h) and 4(i)).

### 3.6. Inhibition of GSDMD Mediates Cardiomyocyte Death through the Depletion of NAD^+^ and ATP

When DNA is damaged, PARP-1 immediately binds to the DNA broken chain and modifies the properties of various proteins involved in DNA repair through covalent action. At the same time, PARP-1 separates from the DNA chain through its own ribosylation, allowing other DNA repair proteins to bind to the broken point of the DNA chain for repair. When DNA damage is severe, excessive PARylation depletes NAD^+^ and ATP, leading to mitochondrial membrane damage and promoting apoptosis. Meanwhile, it also stays at DNA chain breaks and prevents other DNA repair proteins from repairing and initiates cell death pathway [28–30].

To verify the mechanism of GSDMD inhibition promoting apoptosis through PARylation, we detected the NAD^+^ and ATP levels of shRNA-GSDMD H9C2 cells during the H/R process. Under normal oxygen conditions, the expression of NAD^+^ and ATP in GSDMD knockdown H9C2 cells had no significant difference. After H 6 h/R 2 h, the expression of NAD^+^ in sh-GSDMD H9C2 cells was decreased, and ATP was significantly decreased than those in sh-NC. After H 12 h/R 2 h, the level of ATP in sh-GSDMD H9C2 cells was decreased than that in sh-NC, and NAD^+^ was almost depleted in all groups (Figures 4(j) and 4(k)). The results showed that GSDMD knockdown promoted the depletion of NAD^+^ and ATP, which decreased hypoxia tolerance and accelerated cell death.

### 3.7. PARP-1 Inhibitor Reduces GSDMD Defect-Mediated Myocardial Injury during I/R

PARP-1 plays a crucial role in regulating the balance between cell damage and repair. When ischemia and hypoxia occur and lead to DNA fragmentation in cardiomyocytes, PARP-1 is activated to guide the repair of damaged DNA through PARylation. However, persistent excessive activation of PARylation promotes depletion of NAD^+^ and ATP, leading to further cell death [31]. PJ34 is a highly effective PARP inhibitor, and its inhibition of PARP-1 activity is mainly through the competitive blocking of NAD^+^ binding sites on the PARP-1 molecule [32, 33].

**Figure 5:**
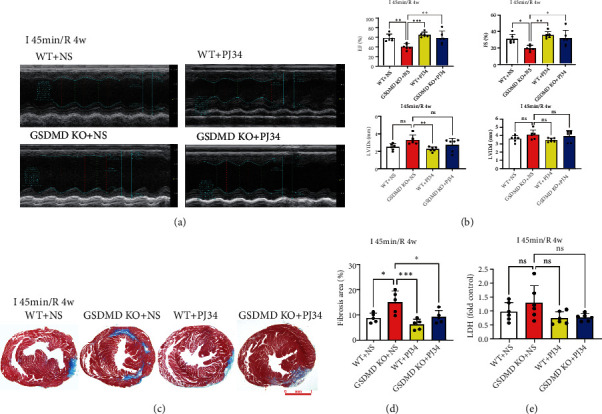
PARP-1 inhibitor reduces GSDMD defect-mediated myocardial injury during I/R. (a) Cardiac function was detected by echocardiography. I 45 min/R 4 w. WT+NS, GSDMD KO+NS, WT+PJ34, and GSDMD KO+PJ34. (b) Quantification of EF, FS, LVIDs, and LVIDd. I 45 min/R 4 w. WT+NS, GSDMD KO+NS, WT+PJ34, and GSDMD KO+PJ34. (c) Myocardial fibrosis was determined by Masson's trichrome staining. I 45 min/R 4 w. WT+NS, GSDMD KO+NS, WT+PJ34, and GSDMD KO+PJ34. Scale bar: 1 mm. (d) Quantification of myocardial fibrosis. I 45 min/R 4 w. WT+NS, GSDMD KO+NS, WT+PJ34, and GSDMD KO+PJ34. (e) Serum LDH concentrations. I 45 min/R 4 w. WT+NS, GSDMD KO+NS, WT+PJ34, and GSDMD KO+PJ34.

Previous studies have shown that PJ34 can protect the damaged myocardium of ischemia in both pig models and H9C2 cells [34, 35]. Our study found that GSDMD deficiency significantly enhanced the PARylation, which would cause myocardial injury in the chronic stage of I/R. Therefore, in further experiments, we investigated whether PJ34 could reduce the negative effects of GSDMD deficiency and magnify the protective effect of GSDMD deficiency on cardiomyocytes. It turned out that the heart function of GSDMD KO mice was significantly decreased in I 45 min/R 4 w, and PJ34 significantly improved the heart function of GSDMD KO mice in EF and FS (Figures 5(a) and 5(b)). The level of myocardial fibrosis in GSDMD KO mice was significantly increased in I 45 min/R 4 w, while PJ34 could partially reduce the level of fibrosis (Figures 5(c) and 5(d)). There was no statistical difference in serum LDH of each group at I 45 min/R 4 w (Figure 5(e)).

**Figure 6:**
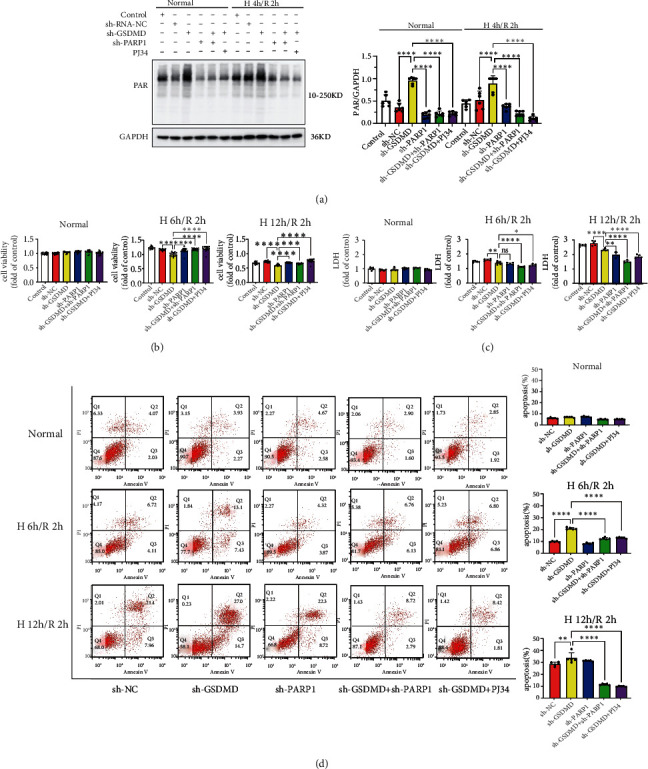
PARP-1 inhibitors reduce GSDMD defect-mediated cardiomyocyte apoptosis. (a) PARylation determined by western blot in control, sh-NC, sh-GSDMD, sh-PARP1, sh-GSDMD+sh-PARP1, sh-GSDMD+PJ34 under normal oxygen, and H 4 h/R 2 h. (b) H9C2 cell viability was detected in control, sh-NC, sh-GSDMD, sh-PARP1, sh-GSDMD+sh-PARP1 and sh-GSDMD+PJ34 under normal oxygen, H 6 h/R 2 h, and H 12 h/R 2 h. (c) LDH release of H9C2 cells was detected in control, sh-NC, sh-GSDMD, sh-PARP1, sh-GSDMD+sh-PARP1 and sh-GSDMD+PJ34 under normal oxygen, H 6 h/R 2 h, and H 12 h/R 2 h. (d) Apoptosis of H9C2 cells was detected by flow cytometry. Normal oxygen, H 6 h/R 2 h, H 12 h/R 2 h. sh-NC, sh-GSDMD, sh-PARP1, sh-GSDMD+sh-PARP1, and sh-GSDMD+PJ34.

Mechanistically, we successfully inhibited PARylation of H9C2 cells by sh-PARP-1 and PJ34 (Figure 6(a)). Further experiments showed that sh-PARP-1 and PJ34 significantly increased the viability of GSDMD-deficient cells after H/R (Figure 6(b)). Meanwhile, sh-PARP-1 and PJ34 also reduced the LDH release of GSDMD-deficient cells after H/R (Figure 6(c)). The results of flow cytometry also showed that sh-GSDMD+sh-PARP-1 or sh-GSDMD+PJ34 could significantly reduce the level of apoptosis compared with sh-GSDMD (Figure 6(d)).

## 4. Discussion

In this study, we demonstrated that knockout of GSDMD alleviates the acute phase of I/R injury by blocking the pyroptosis but aggravates the chronic phase of I/R injury by binding with PARP-1 and activating the PARylation-associated apoptosis pathway. GSDMD and GSDMD-N protein expression was significantly upregulated in I/R heart tissue. Knockout of GSDMD reduced myocardial injury after 45 min ischemia, followed by 24 h reperfusion. However, knockout of GSDMD aggravated myocardial injury after 45 min ischemia, followed by 4 w reperfusion. The opposite effect on the long-term cardiac function in I/R was mediated by PARP-1. These findings revealed a distinct pathway of action for GSDMD in the clinical therapy of myocardial I/R.

Pyroptosis is an inflammatory death characterized by membrane perforation mediated by GSDMD [36]. Previous studies have confirmed that pyroptosis pathway proteins NLRP3 and caspase-1 are involved in AMI and I/R [13, 14]. However, the role of pyroptosis executive protein GSDMD in myocardial ischemia injury is less reported. Recently, Shi et al. found that GSDMD and GSDMD-N were significantly increased in mouse heart tissue and human peripheral blood samples of I/R, and knockout of GSDMD could reduce myocardial injury [20]. Hou et al. found that the level of GSDMD was significantly upregulated in the myocardial tissue of the AMI rat model [37], while Ye et al. reported that the level of GSDMD-N was significantly increased in I/R rats [38]. Lei et al. reported that the level of GSDMD and active subunit GSDMD-N in H9C2 cells significantly increased after oxygen-glucose deprivation [39]. Although the expression of caspase-1/11 and IL-1*β*/IL-18 is controversial due to the differences in subjects and the time of ischemia, consistent with our study, the expression of GSDMD and GSDMD-N increased during myocardial ischemia. It indicated that the GSDMD-mediated pyroptosis pathway is indeed involved in myocardial ischemia infarction. As an executive protein of pyroptosis, GSDMD deficiency prevents cell destruction and releases inflammatory mediators, thereby ameliorating myocardial infarction. In addition, our experiments turned out that GSDMD KO mice showed a significant decrease in myocardial injury in the acute phase of I/R (45 min/24 h), but, contrary to expectations, decreased cardiac function and increased myocardial fibrosis were observed during the chronic phase (45 min/4 w). Unlike previous studies, we first discovered the negative role of GSDMD defect in I/R and its regulation of PARylation.

In the process of myocardial I/R, multiple forms of cell death are involved, among which apoptosis is an important factor that cannot be ignored, and even the main form of myocardial cell death after reperfusion. Therefore, we further investigated the activation of apoptosis regulated by GSDMD KO, and the results showed that GSDMD deficiency could activate caspase-3, caspase-7, and caspase-8 in both mice and cellular I/R models, which were consistent with the research results of GSDMD in macrophages [12]. These results suggested that the inhibition of GSDMD may also have the effect of activating apoptosis during myocardial ischemia injury.

At present, most studies on the molecular role of GSDMD are only considered to be the executive protein of the pyroptosis pathway, and the mechanism of GSDMD regulating apoptosis still needs to be further explored. In this study, we analyzed the interaction proteins of GSDMD in cardiomyocytes and found that there was an interaction between GSDMD and PARP-1. The primary function of PARP-1 is to complete DNA repair through PARylation [40]. In our study, we found that although the active subunit of PARP-1 was not increased, PARP-1-mediated protein PARylation was significantly increased in GSDMD KO mice. According to previous studies, myocardial ischemia and hypoxia activate PARylation, leading to energy depletion and ultimately cell death [18, 24, 38]. In this case, we further analyzed the effect of GSDMD deficiency on the energy regulation of cardiomyocytes. It indicated that there was no statistical difference in the expression of NAD^+^ and ATP among the groups under normal oxygen, but the NAD^+^ of H9C2 cells with GSDMD inhibited was significantly decreased with hypoxia time, and the ATP expression significantly decreased until exhaustion. This suggested that GSDMD deficiency had a minor effect on cardiomyocytes under sufficient energy supply but accelerated the depletion of NAD^+^ and ATP in cardiomyocytes under H/R and eventually led to cell death.

Inhibition of GSDMD protects against I/R injury, but when energy supply is insufficient, the excessive PARylation gradually causes depleted energy and leads to explosive cell death. Based on the findings of this study, we speculated that PARP-1 inhibitors could reduce cell death caused by GSDMD deficiency. PJ34 is a novel PARP-1 inhibitor that inhibits PARylation of the protein by competitively blocking the NAD^+^ binding sites on the PARP-1 [41, 42]. Previous studies have reported that PJ34 protects the heart from I/R injury [34, 35]. In this study, we found that PJ34 in GSDMD KO mice protected cardiac function and reduced myocardial fibrosis in the chronic phase of I/R. On the other hand, MTT and flow cytometry demonstrated that both PJ34 and sh-RNA-PARP-1 effectively reduced cell death caused by GSDMD deficiency.

**Figure 7:**
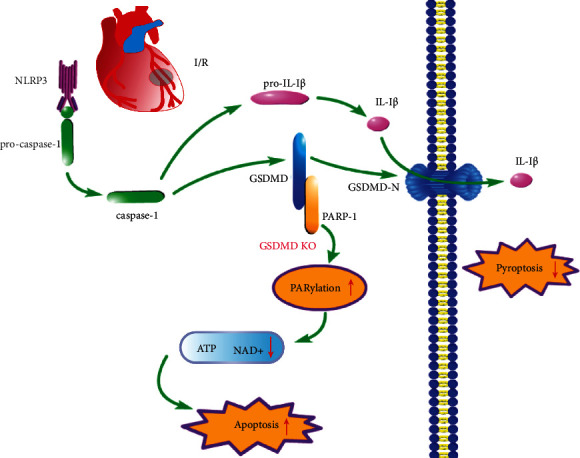
Myocardial I/R activates pyroptosis through the NLRP3-caspase-1-GSDMD pathway. Knockout of GSDMD inhibits pyroptosis but simultaneously activates PARylation, leading to depletion of NAD^+^ and ATP and promoting apoptosis.

In conclusion, we found a distinct function of GSDMD in I/R independent of pyroptosis; that is, inhibition of GSDMD activated PARylation and promoted cardiomyocyte apoptosis (Figure 7). Inhibition of GSDMD improved pyroptosis-mediated myocardial injury in the acute phase of I/R, but excessive PARylation promoted more apoptosis in the chronic phase of I/R. The findings indicate that modulation of GSDMD and PARP-1 interaction provides a novel strategy for clinical treatment of myocardial I/R.

## Data Availability

The data of this study are available from the corresponding authors upon reasonable request.

## Supplementary Materials

Supplementary Materials Figure S1: the efficiency of GSDMD knockout mice and GSDMD shRNAs was validated by western blot. (A) The efficiency of GSDMD knockout in mice. Sham and I 45 min/R 4 h. WT vs. GSDMD KO. (B) The efficiency of GSDMD shRNAs in H9C2 cells. Control, sh-NC, sh-GSDMD1, and sh-GSDMD2 under normal oxygen and H 4 h/R 2 h. Figure S2: expression levels of pyroptosis-associated proteins in GSDMD KO mice. Sham and I 45 min/R 4 h. WT vs. GSDMD KO. Figure S3: (A) the image of Coomassie brilliant blue after Co-IP by GSDMD antibody was used for COIP-MS. (B) COIP-MS analysis of PARP-1. Figure S4: Co-IP assay between GSDMD and PARP-1 was repeated.
