# Supplementary material for: Inhibition of GSDMD Activates Poly(ADP-ribosyl)ation and Promotes Myocardial Ischemia-Reperfusion Injury
Source: Oxid Med Cell Longev. 2022 Jun 24;2022:1115749. doi: 10.1155/2022/1115749 (PMC9249530; doi:10.1155/2022/1115749)
Supplement: Supplementary Materials — Figure S1: the efficiency of GSDMD knockout mice and GSDMD shRNAs was validated by western blot. (A) The efficiency of GSDMD knockout in mice. Sham and I 45 min/R 4 h. WT vs. GSDMD KO. (B) The efficiency of GSDMD shRNAs in H9C2 cells. Control, sh-NC, sh-GSDMD1, and sh-GSDMD2 under normal oxygen and H 4 h/R 2 h. Figure S2: expression levels of pyroptosis-associated proteins in GSDMD KO mice. Sham and I 45 min/R 4 h. WT vs. GSDMD KO. Figure S3: (A) the image of Coomassie brilliant blue after Co-IP by GSDMD antibody was used for COIP-MS. (B) COIP-MS analysis of PARP-1. Figure S4: Co-IP assay between GSDMD and PARP-1 was repeated. [file 1115749.f1.docx]

Fig. S1


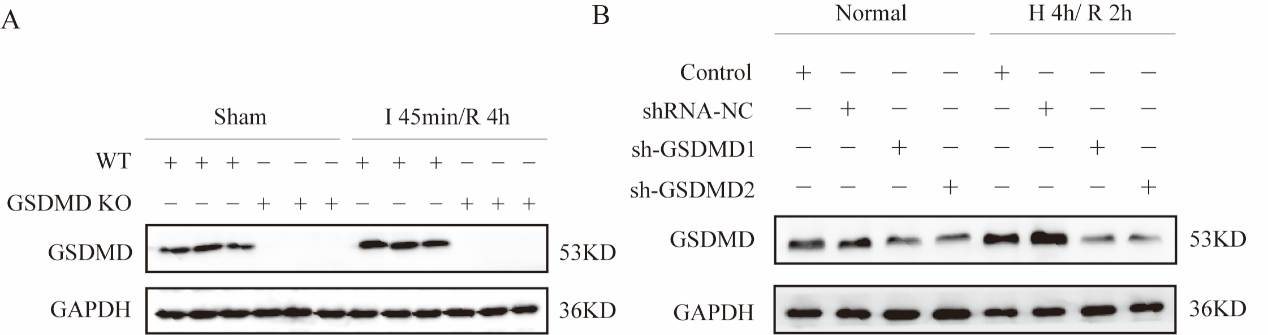


Fig. S2


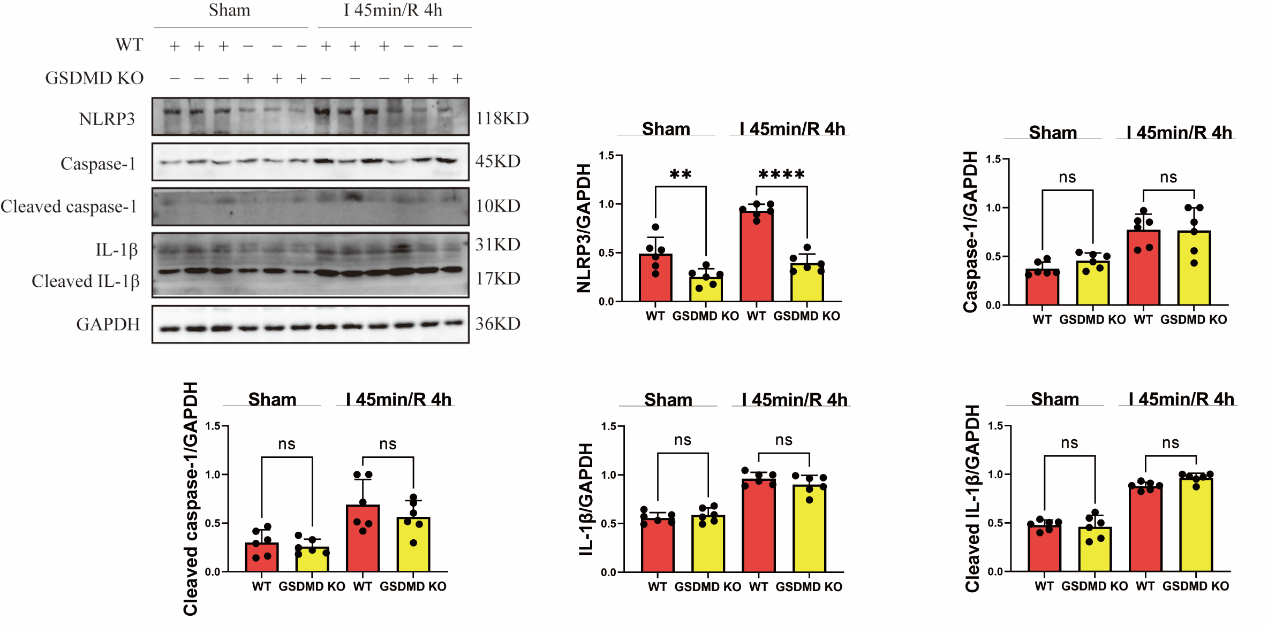


Fig. S3


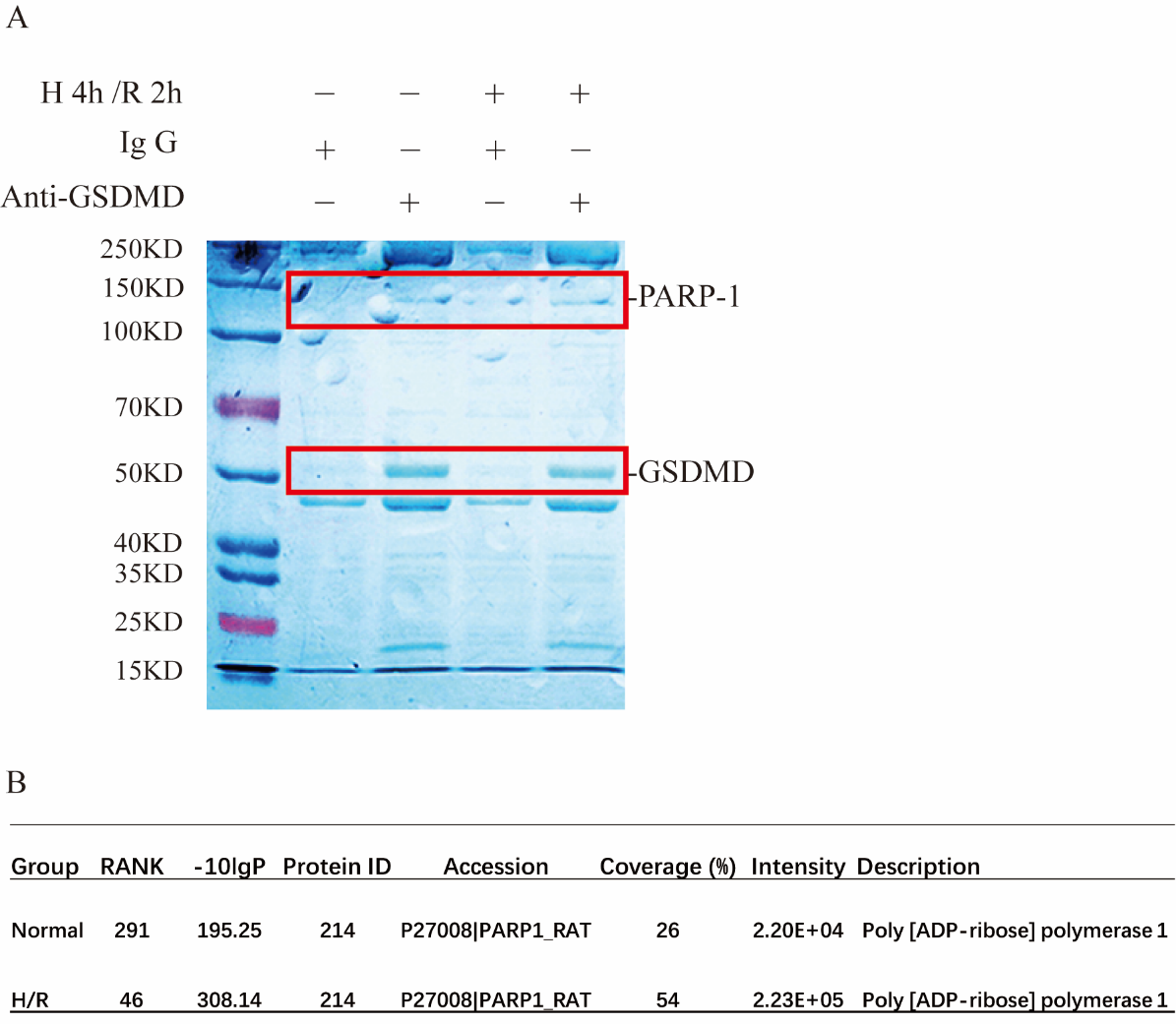


Fig. S4


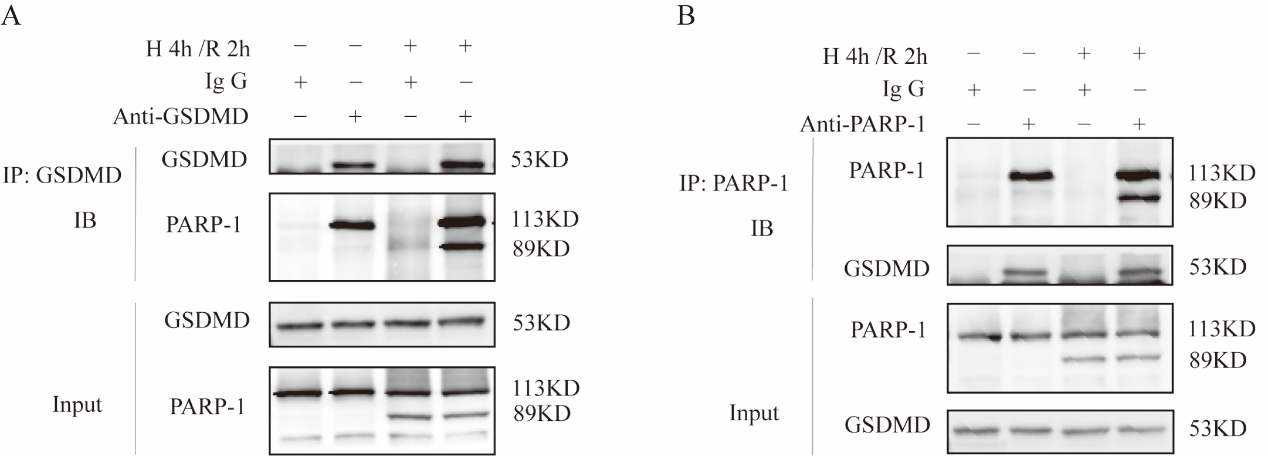


**Supplementary Materials**

Fig. S1 The efficiency of GSDMD knockout mice and GSDMD shRNAs were validated by western blot. (A) The efficiency of GSDMD knockout in mice. Sham and I 45min/R 4h. WT vs GSDMD KO. (B) The efficiency of GSDMD shRNAs in H9C2 cells. Control, sh-NC, sh-GSDMD1, sh-GSDMD2 under normal oxygen and H 4h/R 2h.

Fig.S2 Expression levels of pyroptosis associated proteins in GSDMD KO mice. Sham and I 45min/R 4h. WT vs GSDMD KO.

Fig. S3 (A) The image of coomassie brilliant blue after Co-IP by GSDMD antibody was used for COIP-MS. (B) COIP-MS analysis of PARP-1.

Fig.S4 Co-IP assay between GSDMD and PARP-1 were repeated.
